# Fine‐needle aspiration cytology of the apocrine variant of epithelial‐myoepithelial carcinoma

**DOI:** 10.1002/dc.24308

**Published:** 2019-08-21

**Authors:** Diandra Perez, Rana Naous

**Affiliations:** ^1^ Department of Pathology SUNY Upstate Medical University Syracuse New York

**Keywords:** apocrine epithelial‐myopeithelial carcinoma, apocrine variant, cytology, fine‐needle aspiration, FNA, oncocytic variant

## Abstract

Epithelial‐myoepithelial carcinoma (EMCa) is a rare neoplasm that most frequently afflicts the parotid gland. Histologically, a dual layer of inner, luminal epithelial cells and outer myoepithelial cells with associated background hyalinization characterize these tumors. Several variants of EMCa have been described, including the more recent description of the apocrine variant. We present here a case of a 71‐year‐old male with a parotid mass diagnosed on FNA as an apocrine epithelial‐myoepithelial carcinoma. To our knowledge, this is the first case report describing the cytomorphologic features of apocrine EMCa on FNA smears.

## 1 INTRODUCTION

The name epithelial‐myoepithelial carcinoma (EMCa) was coined by Donath et al. in 1972 but this neoplasm was likely previously reported under other names.^1^ EMCa is a biphasic malignant tumor composed of epithelial and myoepithelial cells. The epithelial cells are luminal and resemble intercalated ductal cells, which is the theorized origin of this tumor.^2^ EMCa is a rare neoplasm comprising approximately 1‐2% of salivary gland neoplasms.^1^, ^3^ These tumors tend to arise in the sixth to seventh decade with a slight predominance in females. It most commonly affects the major salivary glands, with the parotid gland being most common overall, but other reported sites include the upper and lower respiratory tract and palate.^1^, ^3^, ^4^, ^5^ This tumor is considered a low‐grade malignancy due to its rare spread to lymph nodes or distant sites and overall portends a favorable prognosis with 5‐year and 10‐year survivals of 94% and 82%, respectively, as reported by Seethala et al.^6^, ^7^ However, recurrences are seen in up to 50% of cases.^4^

One of the rare variants of classic EMCa is the apocrine variant, which was recently described by Seethala et al.^7^ Herein, we report a case of apocrine EMCa presenting as a parotid mass and diagnosed on FNA. To our knowledge, this is the first case report describing the cytomorphologic features of apocrine EMCa on FNA smears.

## 2 CASE DESCRIPTION

**Figure 1:**
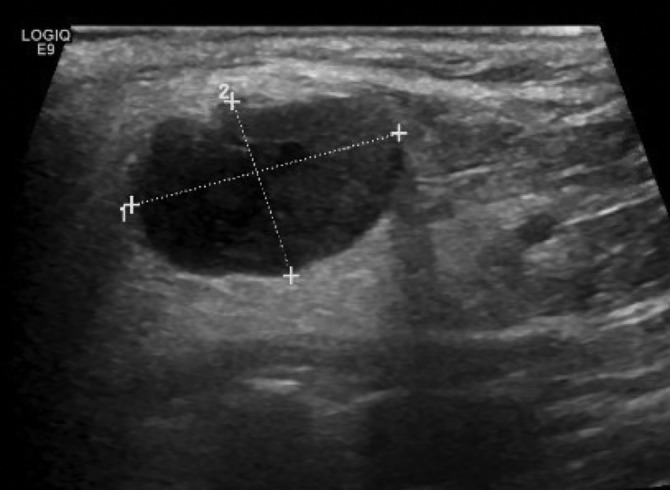
Ultrasound of parotid mass

**Figure 2:**
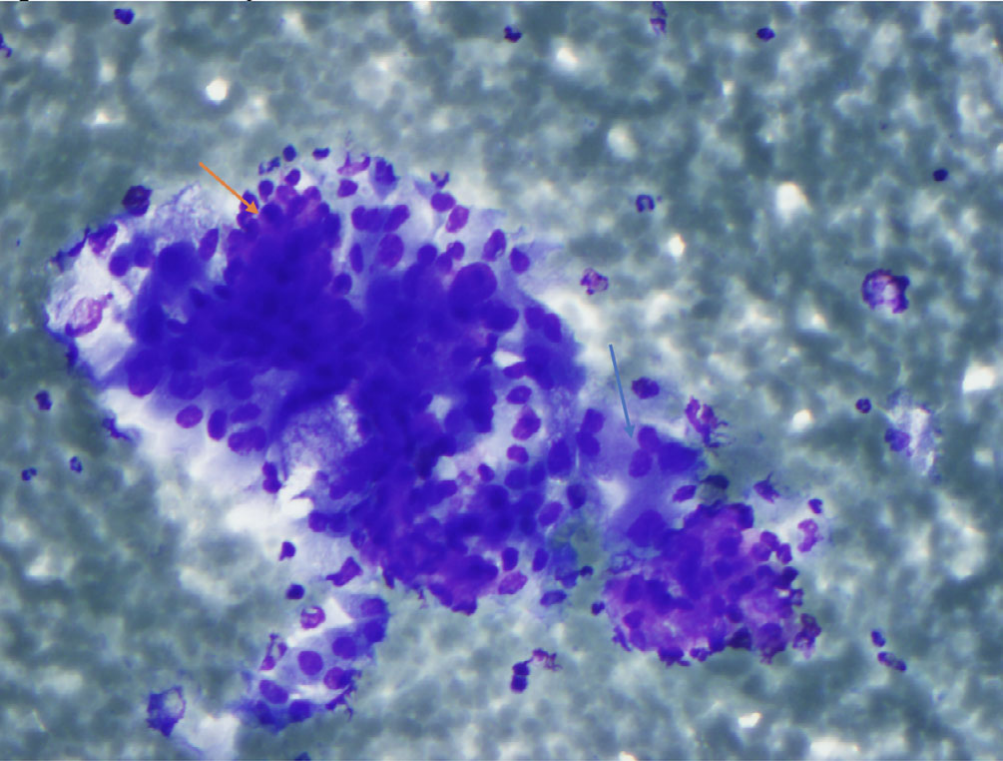
Diff–Quik stain showing the biphasic population of apocrine epithelial cells (blue arrow) admixed with smaller spindled myoepithelial clusters (orange arrow) without hyaline stromal material (×20) [Color figure can be viewed at http://wileyonlinelibrary.com]

**Figure 3:**
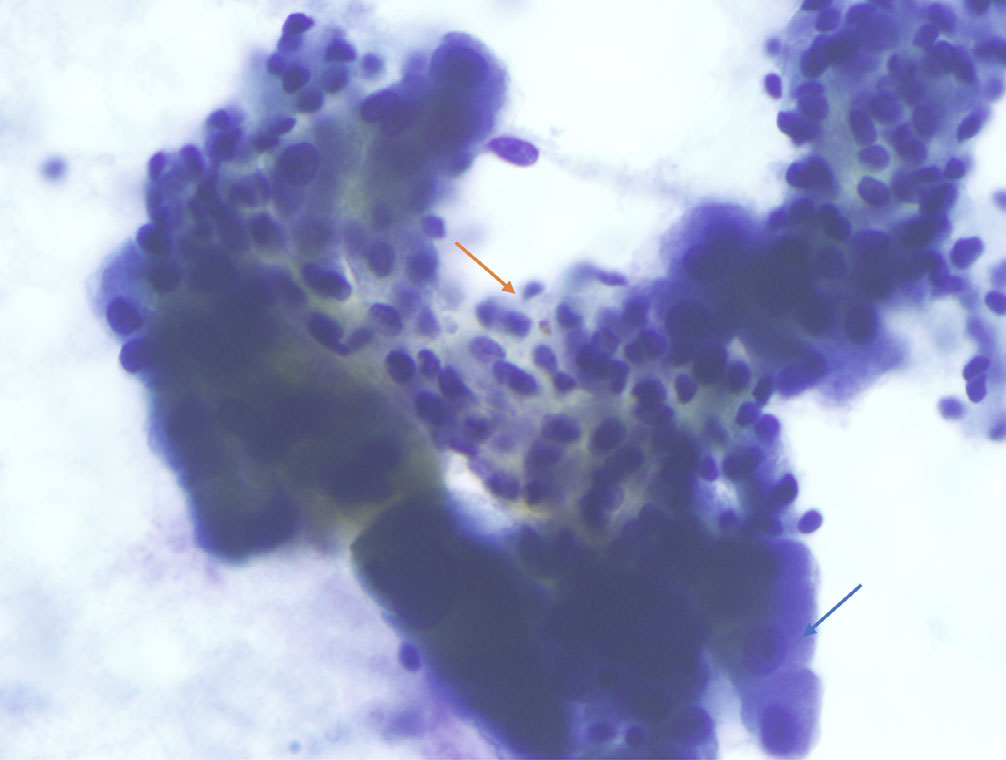
Papanicoulau stain showing the biphasic population of apocrine epithelial cells (blue arrow) admixed with smaller spindled myoepithelial clusters (orange arrow) without hyaline stromal material (×20) [Color figure can be viewed at http://wileyonlinelibrary.com]

**Figure 4:**
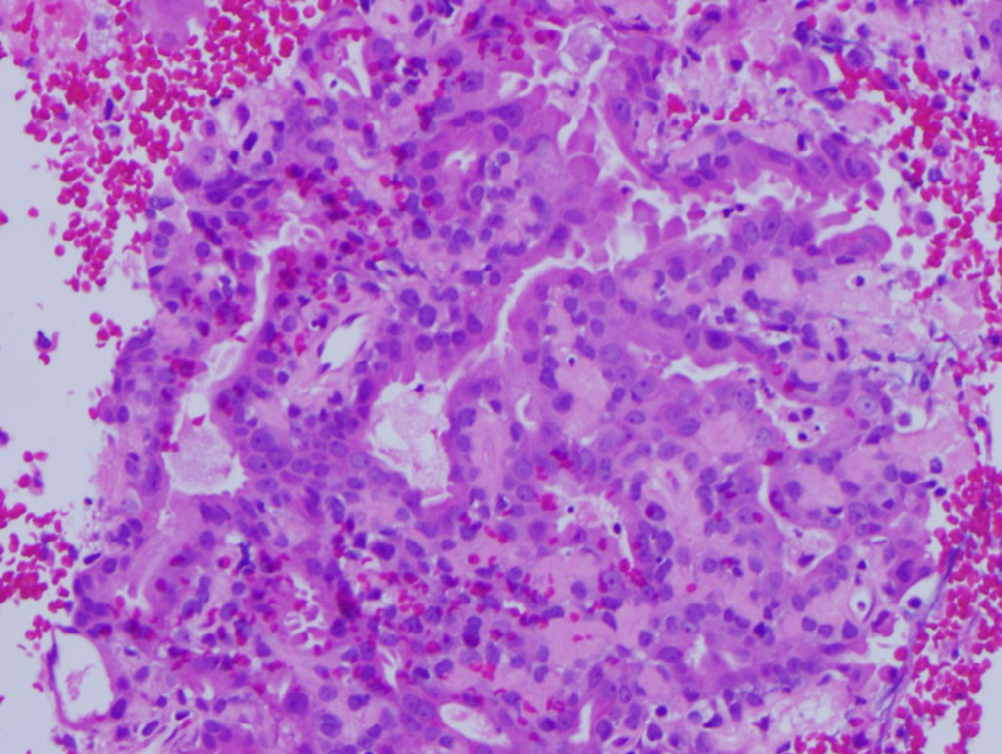
Cell block representing the biphasic population of epithelial‐myopeithelial cells as well as scant dense hyaline stroma (H&E, ×20) [Color figure can be viewed at http://wileyonlinelibrary.com]

**Figure 5:**
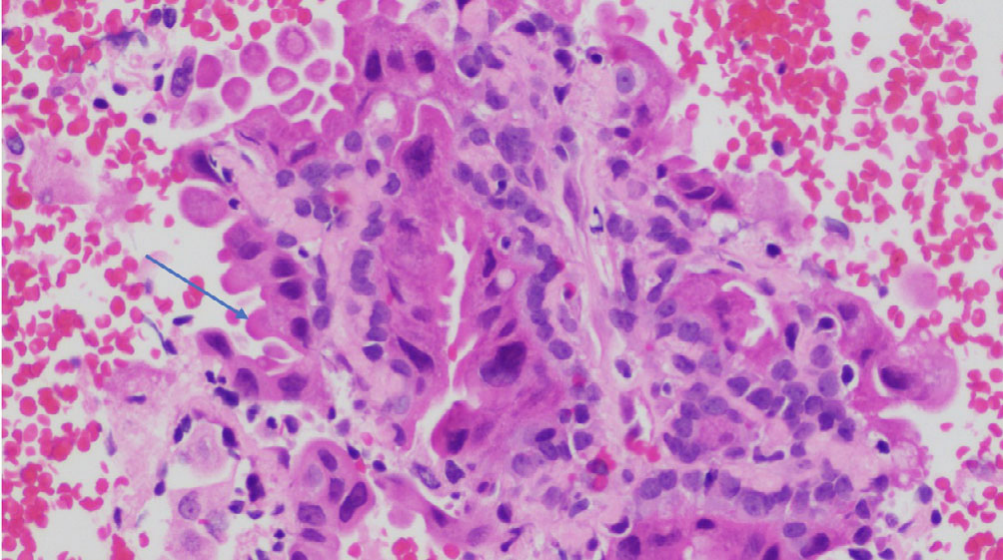
Cell block shows apocrine epithelial cells with apical snouts (blue arrow) (H&E, ×20) [Color figure can be viewed at http://wileyonlinelibrary.com]

**Figure 6:**
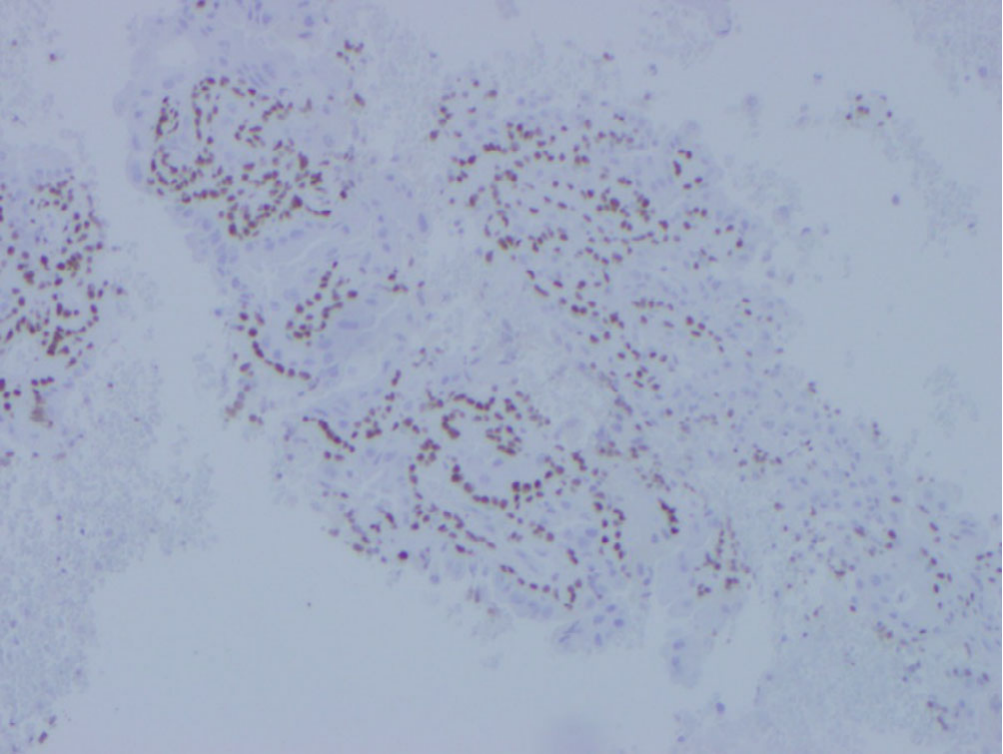
p63 stain highlighting the myoepithelial cells and sparing the apocrine cells (cell block, ×10) [Color figure can be viewed at http://wileyonlinelibrary.com]

**Figure 7:**
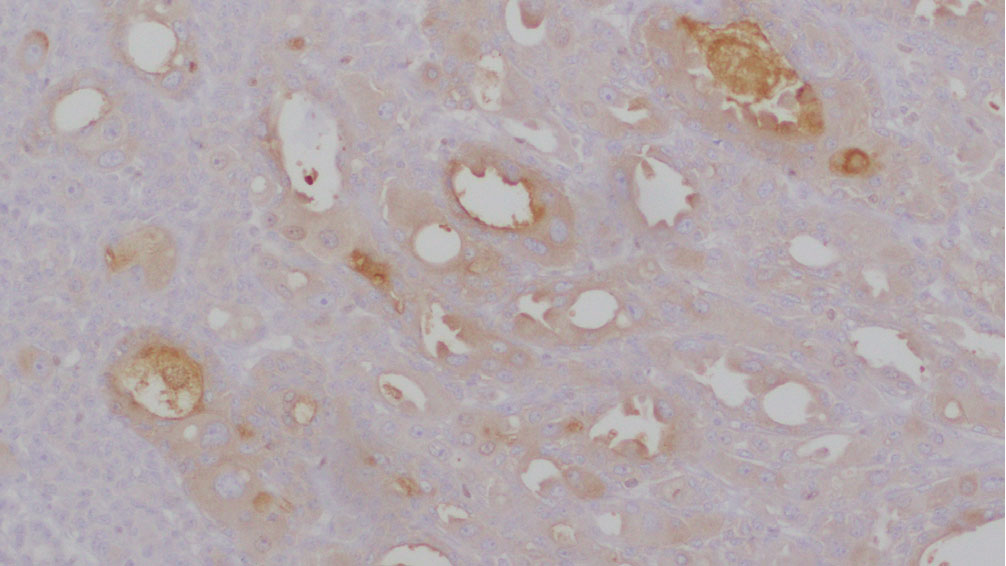
GCDFP15 staining the apocrine cells (cell block, ×10) [Color figure can be viewed at http://wileyonlinelibrary.com]

A 71‐year‐old male smoker with a past medical history of resected bladder carcinoma presented with a right parotid mass noted 4 months prior. CT imaging revealed a 2.5 × 1.9 × 1.8 cm mass without lymphadenopathy. An ultrasound‐guided FNA (Figure 1) of the mass was performed and cytologic examination revealed a biphasic population of numerous apocrine cells arranged singly and in groups with abundant granular cytoplasm, enlarged nuclei, and prominent macronucleoli admixed with a second population of basaloid cells (Figures 2 and 3). The two populations were distributed within absent to scant background hyaline stroma. The cell block showed similar features and demonstrated occasional apical snouts characteristic of apocrine cells (Figures 4 and 5). Immunohistochemical stains performed on the cellblock were positive for p63 (Figure 6), GCDFP15 (Figure 7), SMA, and S100 in the basaloid layer, consistent with its myoepithelial nature. GATA‐3 and mammaglobin highlighted the apocrine layer. Given the overall findings, including the biphasic nature of the lesion and the presence of a prominent myoepithelial layer that does not appear to blend with the background stroma, an apocrine epithelial‐myoepithelial carcinoma were highly suspected.

**Figure 8:**
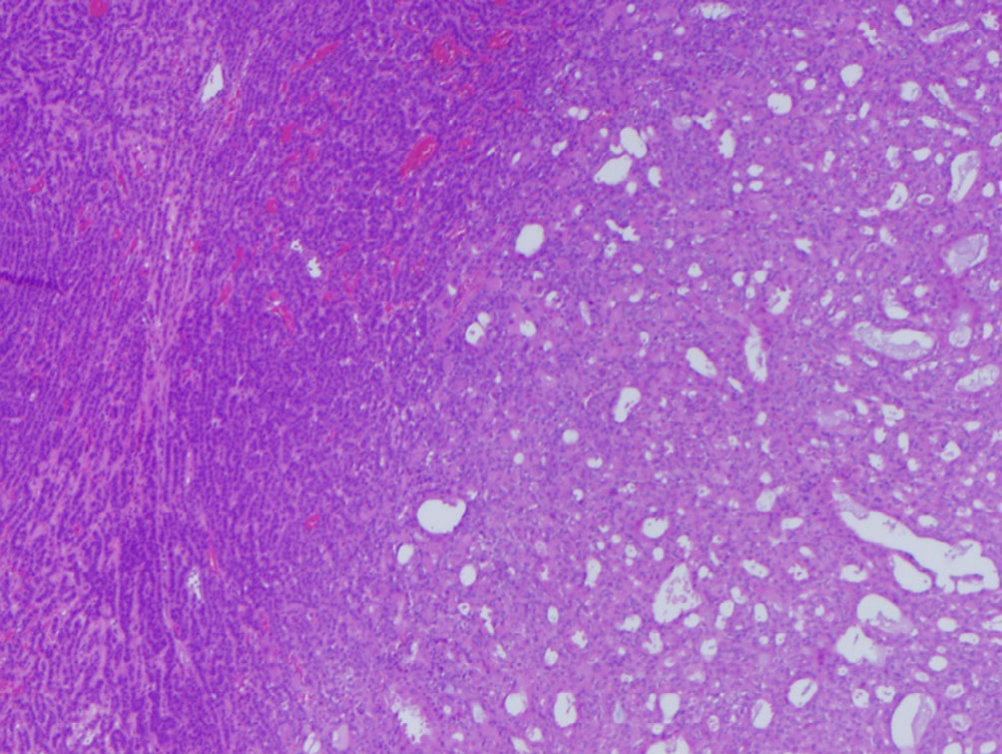
Low power view demonstrating the transition between the solid myoepithelial and the apocrine epithelial‐myoepithelial component (H&E, ×4) [Color figure can be viewed at http://wileyonlinelibrary.com]

**Figure 9:**
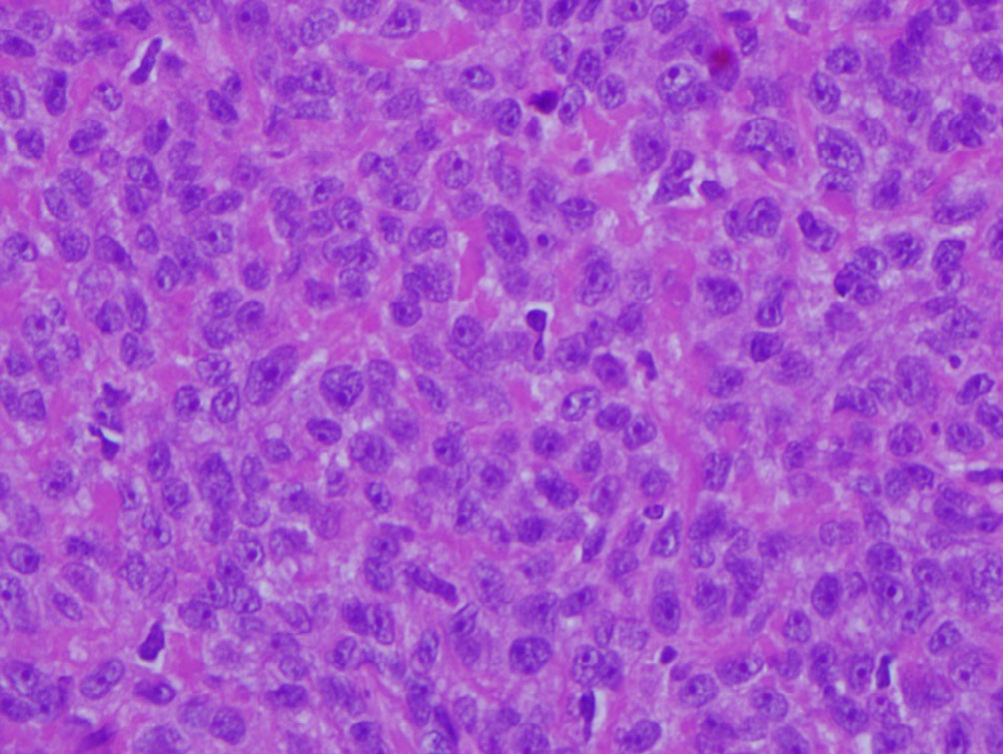
Anaplastic areas within the myoepithelial component, characterized by nuclear pleomorphism, hyperchromasia, and prominent nucleoli (H&E, ×40) [Color figure can be viewed at http://wileyonlinelibrary.com]

**Figure 10:**
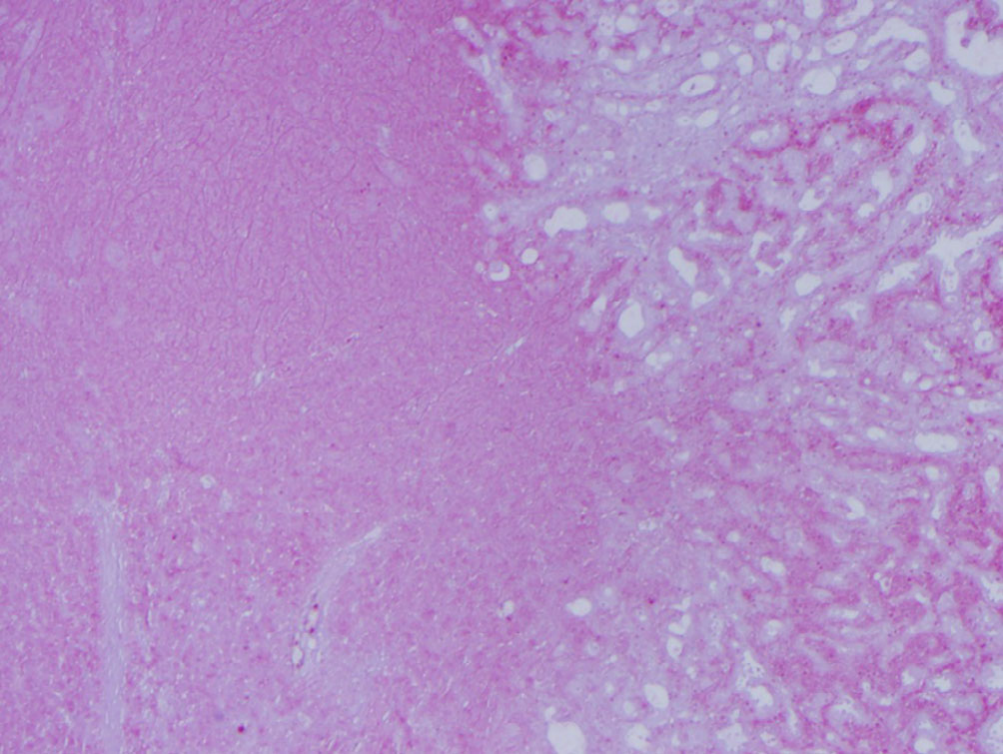
S100 staining myoepithelial cells with sparing of the apocrine component (×4) [Color figure can be viewed at http://wileyonlinelibrary.com]

The patient underwent right superficial parotidectomy. Pathologic examination revealed a 3.8 × 1.9 × 1.7 cm ovoid, ill‐defined, fleshy, pink‐gray lesion which histologically revealed a biphasic salivary gland tumor characterized by apocrine epithelial ductal change closely admixed with myoepithelial cells with varying degree of nuclear atypia (Figure 8). Solid areas of myoepithelial cells with focal anaplastic change were noted (Figure 9). Immunohistochemical stain for S100 highlighted the myoepitheial cells with sparing of the apocrine epithelial ductal cells (Figure 10). The overall findings were consistent with apocrine epithelial‐myoepithelial carcinoma with myoepithelial anaplasia, which is a variant of classic EMCa with a higher recurrence rate than the classic type.^8^

## 3 DISCUSSION

Cytologically, classic EMCa is a tumor with a high false‐negative rate due to its variable morphology and similarity to more common salivary gland lesions.^9^, ^10^ FNA will show a cellular smear with a biphasic population composed of small, basaloid epithelial ductal cells and larger, pale to clear, myoepithelial cells. These larger myoepithelial cells are fragile and leave behind many naked nuclei. Abundant stromal hyaline material can often be seen. There are many diagnostic predicaments when it comes to interpreting EMCa on cytology. The unique and multiple architectural patterns with background hyalinization^3^ classically seen histologically is not maintained on FNA smears, making it nearly impossible to see the bilayered appearance of cells. To complicate this even further, the epithelial and myoepithelial cells can often be difficult to differentiate from one another due to their bland nuclear features and fragility. Additionally, one of the cell populations may not be apparent at all due to sampling, making the critical identification of the biphasic nature impossible.

Recently, an apocrine variant of EMCa has been described by Seethala et al.^7^ This variant is morphologically similar to the oncocytic variant of EMCa, which was first described by Savera et al.^11^ and tends to occur in patients that are a decade older than classic EMCa. Both variants combined make up approximately 8% of EMCa cases.^6^, ^7^ Morphologically, both the apocrine and oncocytic variants maintain the biphasic appearance of classic EMCa. The epithelial component in apocrine EMCa may be arranged in a cribriform or solid pattern, and its nuclei are often large vesicular with prominent nucleoli and abundant granular eosinophilic cytoplasm. The myoepithelial component is often comparable to that of classic EMCa, maintaining its pale to clear cytoplasm. Variable stromal hyaline material may be seen. The apocrine variant is uniquely immunostained by androgen receptor and GCDFP15. In the oncocytic variant, both the epithelial and myoepithelial cells can appear oncocytoid, making the biphasic nature difficult to appreciate. The overall architecture is often papillary with calcifications and occasional sebaceous component.^7^, ^8^ The apocrine variant may look oncoytoid at first glance due to its eosinophilic cytoplasm, but the presence of apical snouts, a feature unique to apocrine EMCa, differentiates it from the smooth luminal epithelial lining characteristic of the oncocytic variant.^8^

Other unique and rare variants of EMCa include EMCa with high grade transformation, EMCa ex pleomorphic adenoma, and sebaceous EMCa.^2^, ^6^, ^7^, ^12^, ^13^, ^14^ EMCa with high grade transformation includes dedifferentiated EMCa and EMCa with anaplasia. Unlike in adenoid cystic carcinoma with high‐grade transformation whereby only the epithelial component undergoes transformation; in EMCa, the high‐grade transformation can be seen in both the epithelial and myoepithelial components.^8^, ^14^

Based on the cytomorphologic features in our case, including the biphasic population with numerous apocrine cells admixed with a second population of basaloid cells within background hyaline stroma, the main differential diagnosis to consider was cellular pleomorphic adenoma with oncocytic/apocrine change. However, the absence of a chondromyxoid stroma and the presence of a distinct myoepithelial layer within the lesion that does not seem to blend into the background stroma made this diagnosis less likely.

Other potential but a less likely entity to consider in the differential diagnosis is salivary duct carcinoma. Apparently, the apocrine component of apocrine EMCa can be confused with the salivary duct carcinoma neoplastic epithelial cells, which tend to have abundant granular cytoplasm that may appear oncocytoid.^15^ To further complicate this issue, rare hybrid cases of EMCa and salivary duct carcinoma have been reported.^12^ Additionally, salivary duct carcinoma arising in a pleomorphic adenoma should also be considered as a possible mimicker, which makes the presence of a distinct myoepithelial layer critical.^8^ Salivary duct carcinoma in situ should be distinguished from EMCa especially as both have a p63‐positive basal cell layer. However, in salivary duct carcinoma in situ, p63 highlights only the residual native basal layer; whereas apocrine EMCa has p63 positivity in both the luminal and basal layer.^8^

Oncocytoma and oncocytic carcinoma aspirates show oncocytes that are indistinguishable from those of oncocytic/apocrine EMCa. However, the absence of p63‐positive myoepithelial cells and the presence of atypia, necrosis, or increased mitotic activity usually points to oncocytic carcinoma. Adenoid cystic carcinoma is also an important diagnostic consideration to keep in mind for oncocytic/apocrine EMCa due to their overlapping morphologic features and the common presence of hyaline stroma. However, the degree of cellular atypia in EMCa is milder than the cytologically atypical adenoid cystic carcinoma.^13^

## 4 CONCLUSION

To our knowledge, there have been no cases reported so far on the cytomorphologic features of the apocrine variant of EMCa diagnosed by FNA. Being a rare entity, apocrine EMCa can be diagnostically challenging when its first presented on cytology smears. Careful cytomorphologic evaluation and a thorough immunohistochemical workup play a vital role in establishing this rare but challenging diagnosis.

## CONFLICT OF INTEREST

No conflicts of interest to disclose.
